# Drug Delivery through the Psoriatic Epidermal Barrier—A “Skin-On-A-Chip” Permeability Study and Ex Vivo Optical Imaging

**DOI:** 10.3390/ijms23084237

**Published:** 2022-04-11

**Authors:** Dorottya Kocsis, Szabina Horváth, Ágnes Kemény, Zsófia Varga-Medveczky, Csaba Pongor, Rózsa Molnár, Anna Mihály, Dániel Farkas, Bese Márton Naszlady, András Fülöp, András Horváth, Balázs Rózsa, Erika Pintér, Rolland Gyulai, Franciska Erdő

**Affiliations:** 1 Faculty of Information Technology and Bionics, Pázmány Péter Catholic University, Práter u. 50a, H-1083 Budapest, Hungary; kocsis.dorottya@itk.ppke.hu (D.K.); varga-medveczky.zsofia@itk.ppke.hu (Z.V.-M.); pongor.csaba.istvan@itk.ppke.hu (C.P.); molnar.rozsa962@gmail.com (R.M.); farkasdaniel0814@gmail.com (D.F.); naszlady.marton.bese@itk.ppke.hu (B.M.N.); fulop.andras@itk.ppke.hu (A.F.); horvath.andras@itk.ppke.hu (A.H.); 2 Department of Dermatology, Venereology and Oncodermatology, Medical School, University of Pécs, H-7624 Pécs, Hungary; horvath.szabina@pte.hu (S.H.); gyulai.rolland@pte.hu (R.G.); 3 Department of Pharmacology and Pharmacotherapy, Medical School, University of Pécs, H-7624 Pécs, Hungary; erika.pinter@aok.pte.hu; 4 Department of Medical Biology, Medical School, University of Pécs, H-7624 Paige, Hungary; 5 Institute of Experimental Medicine, H-1094 Budapest, Hungary; mihalyanna94@gmail.com (A.M.); rozsabal@femtonics.eu (B.R.)

**Keywords:** psoriasis, skin permeability, skin-on-a-chip diffusion cells, TEWL, imiquimod, TRPA1 knock out, TRPV1 knock out, scanning electron microscopy, epidermal barrier

## Abstract

Psoriasis is a chronic inflammatory disease with unmet medical needs. To clarify potential therapeutic targets, different animal models have been developed. In the current study, imiquimod-induced psoriasiform dermatitis was used for monitoring the changes in skin thickness, transepidermal water loss, body weight, blood perfusion and drug permeability for a topical cream formulation of caffeine, both in wild type and in knock out mice. Morphological characterization of control and diseased tissues was performed by scanning electron microscopy and two-photon microscopy. The chemically induced psoriatic group showed increased skin permeability for the model drug during disease progression. In wild type and TRPA1 KO mice, however, enhanced skin thickness and hyperkeratosis blocked further increase of drug penetration at the late phase (96 h). These results indicate that topical drug therapy can be more effective in early phases of plaque development, when skin thickness is lower. Although paracellular connections (tight junctions) are looser in the advanced phase, hyperkeratosis blocks drug delivery through the transappendageal routes. Novel drug formulations may have the potency for effective drug delivery across the epidermal barrier even in the advanced phase. For development of more effective topical drugs, further research is proposed to explore drug penetration both in healthy and diseased conditions.

## 1. Introduction

Psoriasis is a chronic immune mediated skin disorder with a complex pathological background. It affects more than 125 million people globally, and represents a significant health care challenge [1,2,3]. Autoreactive T cells and inflammatory cytokines, especially IL-23 and IL-17, are central to its pathogenesis.

Modeling psoriasis in vivo remains challenging because the disease does not occur naturally in laboratory animals. Specific aspects of its complex immuno-pathology, however, can be studied in drug-induced rodent models or in spontaneous mutation, transgenic and knockout animals, or by using xenotransplantation and immunological reconstitution [4,5,6]. The imiquimod-induced psoriasiform dermatitis model has been one of the most widely used psoriasis model systems recently [7,8,9]. The imiquimod (IMQ)-induced dermatitis model recapitulates several psoriasis-like epidermal, vascular and immune changes, such as parakeratosis, hyperproliferation, induction of MHC II, increased vascularity, vasodilation, and changes in neutrophils, γδ T cells, dendritic cells, plasmacytoid dendritic cells, and Langerhans cells [4,10,11,12]. The upregulation of several psoriasis related cytokines (IL-1β, TNFα, IL-22, IL-23, IL-17) has also been described [9].

Transient Receptor Potential Vanilloid 1 (TRPV1) and Ankyrin 1 (TRPA1) are nonselective cation channels and members of the TRP superfamily serving as multisteric receptors activated by various exo- and endogenous ligands, temperature or pH changes. Both TRPV1 and A1 are expressed on several cell types but were first discovered on sensory neurons, where they co-localize in ~67% of small-diameter sensory nerve endings [13,14]. TRPV1 and A1 are also expressed on numerous resident skin cells, such as keratinocytes, melanocytes and mast cells [15,16] and have a significant role in several physiological processes of the skin [17]. As shown recently by others and our group, TRPV1 and TRPA1 have previously been shown to also play important roles in pathological skin conditions, such as pruritus [18,19], atopic dermatitis [20] and psoriasis [9,21,22]. TRPV1 function seems to be essential for the development of psoriasiform dermatitis—therefore, blocking TRPV1 leads to severely impaired inflammation in the IMQ model. TRPA1, on the other hand, exerts a protective role, and blocking or knocking out TRPA1 enhances the IMQ induced dermatitis.

While several aspects of IMQ-induced dermatitis have been analyzed (including skin thickness, blood perfusion, semiquantitative histopathological evaluation, determination of scaling score to monitor psoriatic symptoms, and collection of spleen and body weight), little data are available on changes in psoriatic skin permeability, and it has not been previously investigated in TRPV1 and TRPA1 knockout mice. Skin permeability is an essential determinant of the epidermal barrier integrity during disease progression, and thus serves as an important factor for the application of topical medications. Epidermal keratinocytes have key roles in maintaining the physical/chemical barrier function of the skin. To execute this aim, keratinocytes perform a continuous differentiation program regulated by a gradual increase of intracellular Ca^2+^ from the basal layer to the outer surface of the epidermis [23,24]. Activation of TRPV1 and the resulting Ca^2+^ influx suppresses cell growth, induces apoptosis [25], and could delay barrier recovery [26]. Administration of the TRPV1 antagonist PAC-14028 accelerated barrier repair [27]. Denda et al. proved a protective effect of TRPA1 in the barrier function of the skin, showing that topically applied TRPA1 agonists with simultaneous cold stimulus increased the rate of skin permeability barrier recovery [28].

In the current study, the drug absorption through the psoriatic epidermal barrier was tested as a function of time during the development of IMQ-induced psoriasiform dermatitis in wild type, TRPV1 KO and TRPA1 KO mice. The model drug for permeability testing was the topically widely used hydrophilic drug caffeine, formulated in a vaseline based suspension cream. The surface properties of the intact, vaseline treated control and imiquimod treated skins were also compared by optical imaging (scanning electron microscopy and two-photon microscopy). The permeability studies were conducted in a “skin-on-a-chip” microfluidic device, which has been developed and validated previously by our laboratory [29,30].

## 2. Results

### 2.1. In Vivo Induction of Psoriasiform Dermatitis

**Figure 1:**
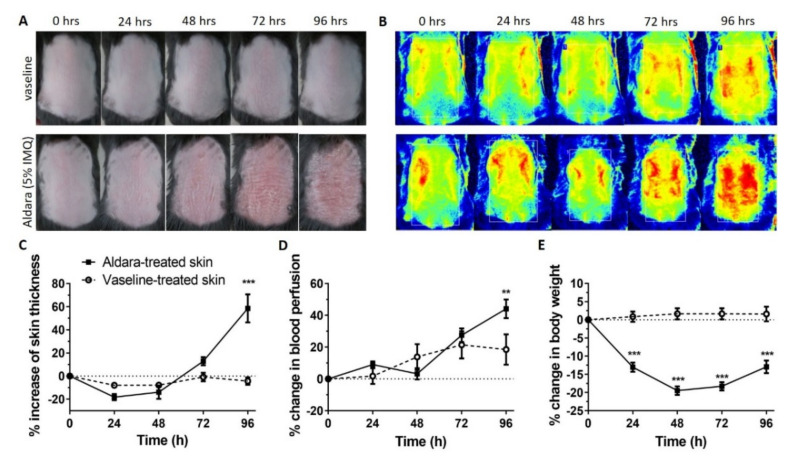
(**A**) Clinical signs of topical Aldara and vaseline treatment on the dorsal skin of C57BL/6J mice on each day of the experiment. (**B**) Representative images of blood perfusion changes on C57BL/6J mice dorsal skin on each day of the experiment induced by topical application of Aldara or vaseline. (blue-green-yellow: low perfusion, red: high perfusion areas). (**C**) Percent change in skin thickness after Aldara or vaseline treatment in C57BL/6J mice. Data are expressed as percent increase of back skin thickness compared with 0 hrs baseline initial values. Data are means ± SEM for *n* = 5/group. *** *p* < 0.001, (**D**) Percent change in dorsal skin blood flow after Aldara or vaseline treatment in C57BL/6J mice. Data are expressed as percentage of blood perfusion change compared to 0 h values. Data are means ± SEM for *n* = 5/group. ** *p* < 0.005, (**E**) Percent change in body weight after Aldara or vaseline treatment in C57BL/6J mice. Data are expressed as percent change of weight compared with 0 h baseline initial values. Data are means ± SEM for *n* = 5/group. *** *p* < 0.001.

The chemical induction of psoriasiform dermatitis was monitored during the 5-day observation period. The clinical signs, the dermal blood perfusion, skin thickness and the body weight changes were measured during the development of the disease. Photos and laser speckle images show a gradial development of redness that is hyperperfusion (Figure 1A–E).

### 2.2. Diffusion Studies

#### 2.2.1. Barrier Desintegration during Progression of Psoriasis

**Figure 2:**
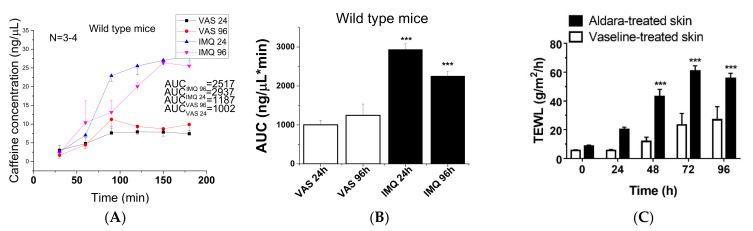
Skin penetration of 2% caffeine cream in wild type C57BL/6J mice: (**A**) concentration-time profiles of vaseline treated controls and imiquimod-treated psoriatic animals, (**B**) AUC values generated from (**A**). The measurements were performed in skin-on-a-chip microfluidic device in triplicates (*n* = 3/group). The data are expressed as means +/− SEM. AUC values were calculated by OriginPro 2015 Software (OriginLab Corporation). ***: *p* < 0.001 vs. vaseline treated controls. (**C**) Transepidermal water loss (TEWL) after Aldara or vaseline treatment in C57BL/6J WT mice. Data are expressed as means ± SEM for *n* = 5/group. *** *p* < 0.001.

The concentration-time profiles of caffeine absorption through the epidermal barrier in vaseline treated control and imiquimod-treated psoriatic animals are presented in Figure 2A,B. The skin absorption of the test drug was monitored for 5 h, but the penetration reached its maximum at 3 h and after that a plateau phase was observed. Therefore in this graphic, only the concentrations measured in the first 180 min are presented. The permeability of mouse skins increased during the progression of psoriasiform dermatitis, but after 96 h a relative reduction (compared to 24 h) can be observed in the drug penetration through the diseased (IMQ treated) skins. Transepidermal water loss (TEWL) was monitored during the 5-day experiment. The impairment of epidermal barrier function became statistically significant 48 h after the first Aldara treatment and remained like that until the end of the experiment (Figure 2C).

A statistically significant inrease can be seen in permeability on psoriatic skins compared to the controls, but a reduced permeability appeared in advanced diseased preparations (96 h). This observation can be explained with the thickening of the stratum corneum during the psoriatic plaque formation (Figure 1C) at a later phase of the disease.

#### 2.2.2. Comparison of Wild Type, TRPA1 KO and TRPV1 KO Knock Out Mouse Strains by Testing Drug Permeability during Progression of Psoriasis

**Figure 3:**
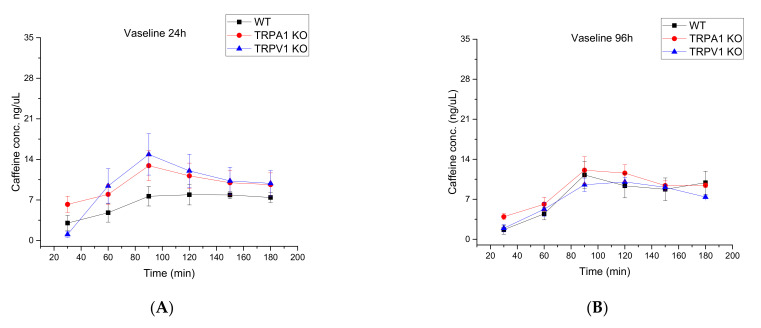

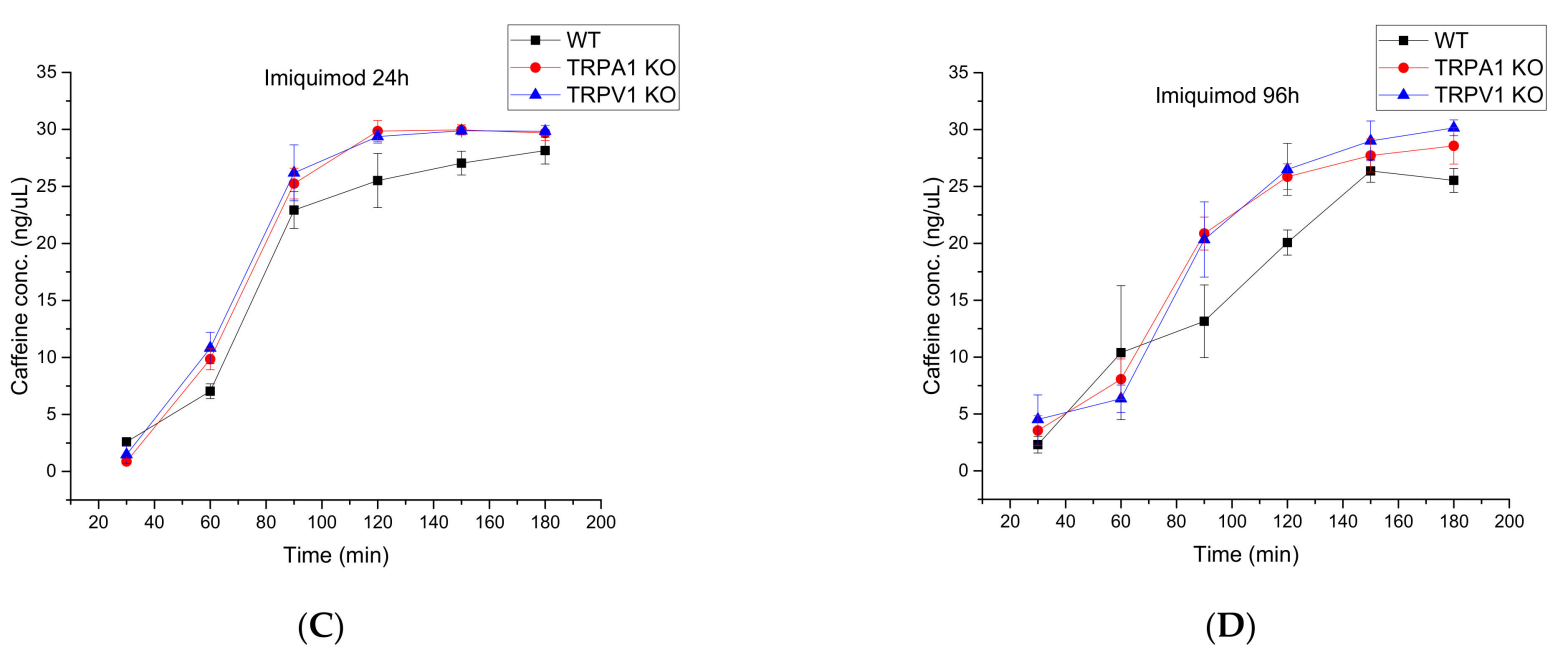
Concentration-time profiles of mouse skin permeability for 2% caffeine cream in C57BL/6J WT mice, TRPA1−/− and TRPV1−/− transgenic mice. (**A**): Vaseline 24 h, (**B**): Vaseline 96 h, (**C**): Imiquimod 24 h, (**D**): Imiquimod 96 h. The measurements were performed in a skin-on-a-chip microfluidic device in triplicates (*n* = 3–4 animals/group). The data are expressed as means +/− SEM. AUC values were generated by OriginPro 2015 Software (OriginLab Corporation, Macasoft Bt, Győr, Hungary).

**Figure 4:**
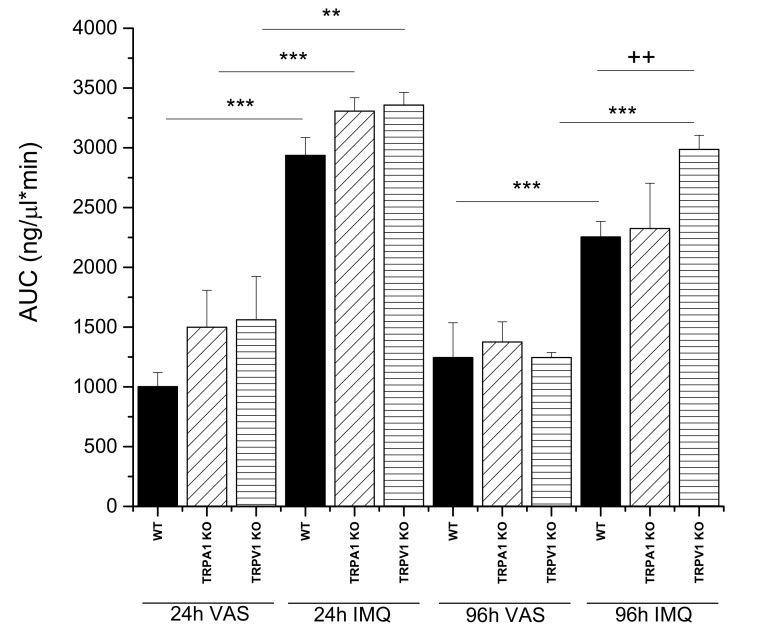
AUC values generated from concentration-time profiles of caffeine absorption from 2% cream formulation through the epidermal barrier for wild type and transgenic mice (TRPA1 KO and TRPV1 KO). Vaseline (VAS) (vehicle)-treated and psoriatic (IMQ) (5% imiquimod-treated) mouse skins were compared. The changes in skin permeability were monitored at two different time points (24 h and 96 h post-treatment). (Means +/− SEM). *N* = 3–4/group. **: *p* < 0.01, ***: *p* < 0.001 vs. vaseline treated controls, ++: *p* < 0.01 vs. 96 h IMQ WT.

In the second series of experiments, the wild type and KO animals were compared by testing drug permeability under healthy and diseased conditions. As shown in Figure 3A–D and Figure 4, the three different strains showed similar time-characteristics in the permeability during the development of psoriasis.

A highly significant difference was demonstrated between the Vaseline-treated and psoriatic groups at both observation points (24 h and 96 h) (Figure 4) for all three mouse strains. However, there was a reduction in the drug permeability between 24 h and 96 h (Figure 4), especially in WT and TRPA1 KO mice. The possible explanation for this phenomenon is the radically increasing thickness of the scaly skin in psoriatic animals between 24 and 96 h (Figure 1C). On the contrary, in the case of TRPV1 KO animals, there was no significant reduction in the permeability at the final stage, indicating a lower degree of inflammation in this strain. This results are in accordance with the literature where the pathological role of TRPV1 was described in WT animals, proving this statement by the protection observed against the psoriatic symptoms in the TRPV1 KO mice [9,21].

The routes of penetration of caffeine across the skin barrier includes the transappendageal pathways, which are partially blocked in the advanced psoriatic skins. Both hair follicles and sweat ducts become clogged in the thickened psoriatic dead epidermis and although the inflammation is progressed (tight junctions in the granular keratinocyte layer are damaged), the drug penetration becomes lower at this late stage of the disease.

### 2.3. Scanning Electron Microscopy (SEM)

**Figure 5:**
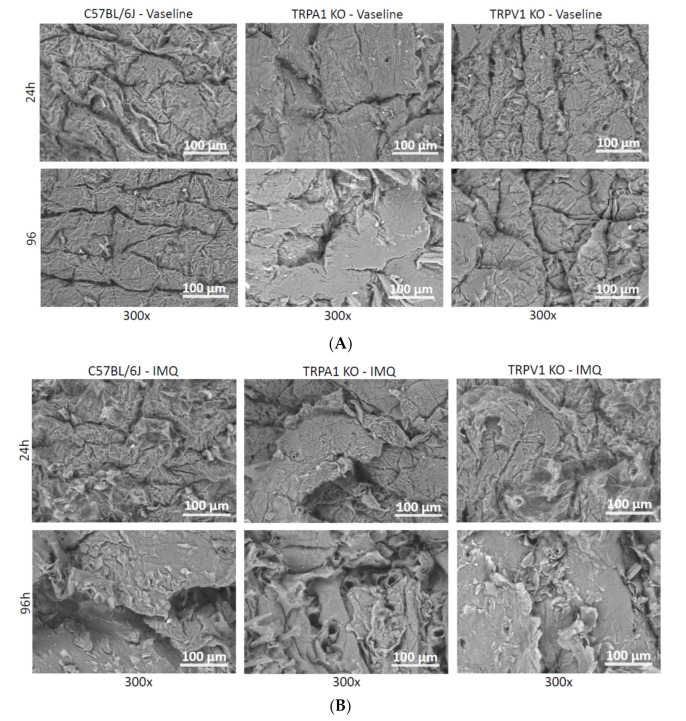
Scanning electron microscopy of vaseline- (**A**) vs. imiquimod (IMQ)- (**B**) treated mouse skins from three different mouse strains (WT, TRPA1 KO and TRPV1 KO). After dissection, the skins were fixed in paraformaldehyde (PFA) and then dehydrated by increasing concentration of ethanol. Finally vacuum drying was applied before the microscopic imaging. Exposure to 5% imiquimod cream resulted in a significant increase in scaly detachment of dead corneocytes at the surface of the stratum corneum, especially at 96 h exposition time.

The surface structures of the vaseline and Aldara treated skins were examined under scanning electron microscopy (Figure 5). There was no significant deviation in the appearance of the vaseline covered skins and intact control tissues. However, in the psoriatic skins, a well detectable morphological difference appeared in all three mouse strains tested. The most prominent characteristic was the detachment of scaly corneocyte plaques, which was visible in TRPA1 mice after 96 h (Figure 5b).

For quantification of the degree of psoriasiform inflammation, a computerized classical image analysis method was performed on the highest magnification scanning electron microscopic pictures (300×) (see at Section 2.4). This type of morphological evaluation has the benefit over conventional histopathology of being a rapid process and SEM in combination with image analysis provides quantitative, objective data characteristic for the disorder.

### 2.4. SEM Image Analysis by Classical Image Processing

During the image analysis, three different samples (WT, TRPV1 KO, TRPA1 KO) were used for each category (psoriatic 24 h and 96 h post-treatment, and vaseline 24 h and 96 h post-treatment) and N_G_ (number of gaps) and N_PC_ (pixel number of the cell regions) values were calculated.

**Figure 6:**
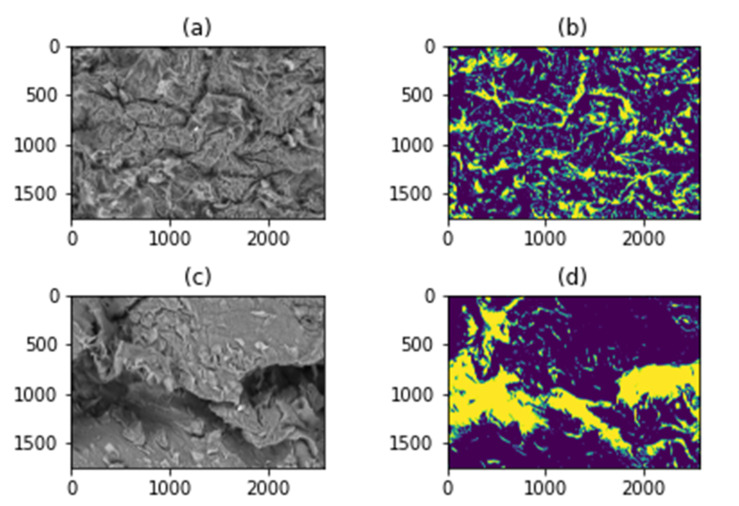
(**a**): The original WT IMQ 24 h scanning electronmicroscopic image; (**b**): the segmented version of WT IMQ 24 h image, yellow indicates the gaps, while blue indicates contiguous cell surfaces. (**c**): the original WT IMQ 96 h microscopic image; (**d**) the segmented version of the WT IMQ 96 h image, yellow indicates the gaps, while blue indicates contiguous cell surfaces.

Figure 6 shows how the algorithm separates gaps and cells; yellow color means the gap, and blue color indicates the cell surfaces.

**Figure 7:**
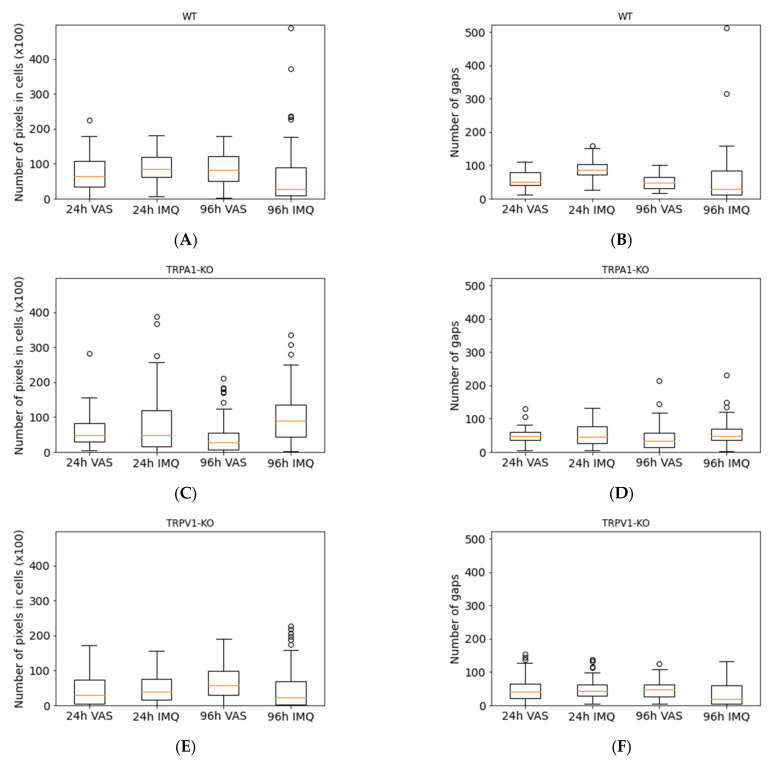
Image analysis of scanning electron microscopic pictures for cell regions (**A**,**C**,**E**) and for gaps between the cells (**B**,**D**,**F**). The figures on the left show the N_PC_ values and on the right show the N_G_ values in box plots of the WT, TRPA1 KO, and TRPV1 KO samples for the different conditions (psoriatic 24 h and 96 h, and vaseline treated controls 24 h and 96 h post-treatment). The images for all strains and conditions were divided into 60 images with a resolution of 250 × 250. Box plots were calculated from 60-image sequences.

The means and standard deviations of 3–3 samples belonging to each category (IMQ 24 h and 96 h, VAS 24 h and 96 h) were calculated. In addition, the boxplots (Figure 7) about N_G_ and N_PC_ parameters were generated. The 300× magnification images in the case of all strains and conditions were divided into 60 images with a resolution of 250 × 250. Boxplots were calculated from 60-image sequences.

The ratio of cells to gaps was examined separately using N_G_ and N_PC_ values in the different strains. For this purpose, the images were divided into resolution of 1750 × 2560 pixels 250 × 250 images, so that a total of 60 images were obtained in each condition. The results (N_G_ and N_PC_ values) are shown in Figure 7 and Table 1. The cell formation and scaly detouchment were the highest in the psoriatic skins of wild type mice at 96 h in accordance with the skin thickness data in Figure 1C. These results were confirmed by the digitalized scanning electron microscopic pictures (Figure 7) by the number of cell regions. The severity of plaque formation was also significant in the psoriatic TRPA1 KO skins both at 24 and 96 h, and seemed to be the less expressed in TRPV1 IMQ skins. The number of intercellular gaps was also the highest in wild type psoriatic animals at 96 h.

### 2.5. Two-Photon Microscopy

**Figure 8:**
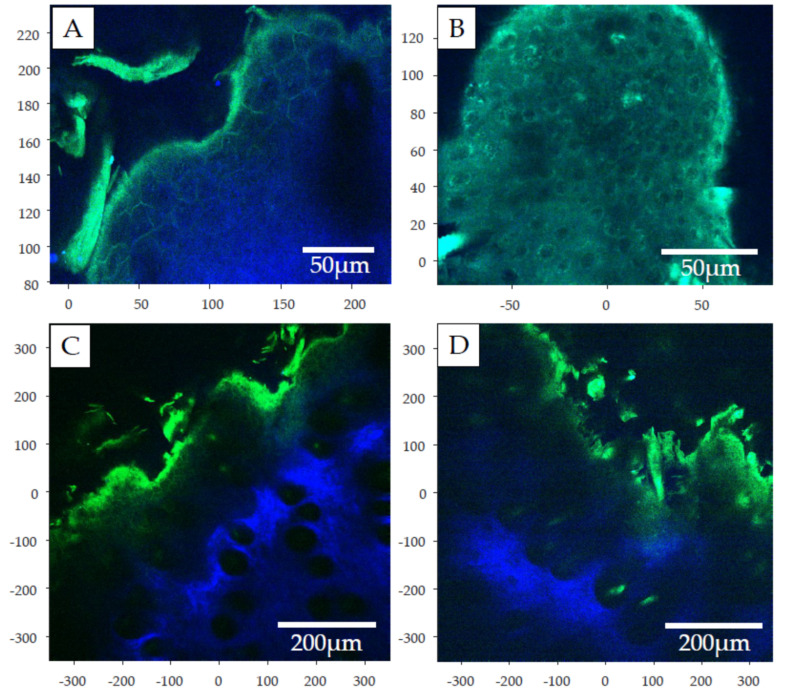
Two-photon images of vaseline-treated (**A**,**C**) and 5% imiquimod-treated (**B**,**D**) mouse skins. The proliferating scaly keratinocytes can be seen in (**A**) and (**B**), while the stratum corneum (green) and subcutaneous adipose tissue (blue) are shown in the cross sectional view on (**C**) and (**D**).

For visualization of the surface and cross sectional structures of psoriatic tissue in wild type animals, two-photon microscopic pictures were generated on the unlabelled tissues at 96 h after the induction of psoriasis. The surface images (Figure 8A,B) show the proliferating keratinocytes in the inflamed tissue, while in the cross sectional views (Figure 8C,D), the subcutaneous adipose tissue also becomes visible in blue color. The images presented here are only for illustration of the psoriatic epidermal process, and used for making a validation of two photon microscopy for utilization in dermatological evaluation. Further studies are planned to continue this kind of analysis of psoriatic skins in a larger number of samples and by combination with image analysis.

The skin samples were not labeled with any exogeneous fluorophores, the fluorescence seen on Figure 8 comes from endogenous fluorophores (autofluorescence) (Table 2). The samples were excited at 900 nm, at this excitation wavelength in rat and human skin samples flavin-adenine-dinucleotides (FADs, λem490–650 nm), keratin (λem450–550 nm) [31], flavoprotein (λem460–480 nm) and lipoamide dehydrogenase (λem510–550 nm) [32] are the main endogenous fluorophores. The used filter cube in the two-photon microscope is able to detect light at 435–485 nm (blue) and 500–550 nm (green). Based on these and the components’ emission wavelength, FADs and keratin can be seen in both the blue and green channel, while flavoprotein can only be seen in blue and lipoamide dehydrogenase only in green.

## 3. Discussion and Conclusions

Different alternatives exist for the treatment of psoriasis in the clinics. These include topical drugs (corticoids, vitamin D derivatives and retinoids) and phototherapy for mild-to-moderate cases, and systemic treatment for severe disease. Drugs used in systemic therapy for psoriasis include both small molecules such as methotrexate (MTX), retinoids, or cyclosporin, and also biologics such as monoclonal antibodies and receptor fusion proteins. Most of the small molecules reduce keratinocyte proliferation or suppress immune activity while the target of biologics is the cascade of inflammatory cytokines/cytokine receptors in the IL-23/Th17 axis and TNF-signaling [33,34]. Despite significant recent advances in the field of systemic antipsoriatic therapy, most psoriasis patients still require some form of topical treatment (either as add-on or as standalone therapy). To increase the success of topical therapy, different new formulations and novel drug delivery strategies have been tested (liposomes, lipospheres, nanostructured lipid carriers, niosomes, nanoemulsions, nanospheres, microneedles, ethosomes, nanocrystals, and foams) [5,15,35]. Calcipotriol/betamethasone diproprionate aerosol foam is a recently developed new psoriasis treatment option that is rapidly effective, has greater efficacy versus ointment and gel formulations, and was shown to improve patient treatment satisfaction [36]. On the other hand, adherence to the traditional ointment formulation remains a significant challenge for psoriatic patients because the daily treatment regimen can be cumbersome and time consuming.

Regarding these diversified therapeutic intervention strategies, the topical delivery route has a major importance in psoriasis therapy. Therefore, it is crucial to study the changes of the epidermal barrier function during the progression of the psoriatic disorder. The skin barrier is composed of two basic elements: (1) a physical barrier, which is primarily localized in the epidermis, and (2) an molecular barrier, which is present both in the dermis and epidermis [37]. These two systems interact cooperatively to maintain skin homeostasis and overall health [38]. However, if one of them, or both are dysregulated, several skin diseases may occur. In addition, psoriasis is associated with disrupted barrier function, the dysregulated differentiation and proliferation of keratinocytes, chronic inflammation and modified chemical composition of the diseased skin [39]. The pathogenesis of psoriasis includes the contribution of keratinocytes, immune cells, genetic and environmental factors. In psoriasis, the physical skin barrier becomes irregular due to hyperkeratosis (the thickening of the stratum corneum) and acanthosis (the thickening of other epidermal layers), generated by the non-inhibited proliferation and the abnormal differentiation of keratinocytes [40]. The permeability data provided by the current study are in accordance with the literature showing worsening of the barrier function of epidermis (higher permeability), and on the other hand showing increasing barrier thickness at the more advanced stage of the psoriatic process in genetically modified mice (TRPA1 KO) (relative reduction in caffeine absorption). Psoriatic plaques have a keratinocyte density that is 2–5 times higher than that of the normal skin [41]. These data were also supported by the image analysis results of our scanning electron microscopic study (Table 1 and Figure 7). Epidermal proliferation markers (Ki-67, cyclin D1, cyclin E, retinoblastoma protein, proliferating cell nuclear antigen and p63) are highly expressed in the psoriatic lesions, but they are reduced following effective treatment as it was reported by different authors [42,43].

The role of TRPV1 and TRPA1 ion channels in epidermal barrier formation was previously investigated by Denda and co-workers. The TRPV1 receptor antagonist capsazepine was able to inhibit a glycolic acid-induced keratinocyte proliferation response and ATP release in a skin equivalent model [44], furthermore activation of TRPV1 by noxious heat delayed skin barrier recovery after tape stripping in hairless mouse and normal human skin [26]. However, TRPA1 activation with two distinct agonists, such as allyl-isothiocyanate or cinnamaldehyde and also with a brief exposure to cold temperature (which is a TRPA1 activator) accelerated the barrier recovery. This TRPA1-mediated beneficial effect was inhibited by the selective TRPA1 antagonist HC030031 [28].

In summary, the current study provided evidence for the increasing drug permeability through the epidermal barrier with the progression of psoriasiform dermatitis in a chemically induced disease model in wild type mice. The previously proved role of TRPA1 and TRPV1 cation channels in the pathomechanism of psoriasis was studied in our skin-on-a-chip diffusion chamber as well, but the deletion of these proteins has not resulted in a statistically significant difference in the transdermal drug delivery relative to WT. Furthermore, in WT and TRPA1 KO mice, the drug permeability showed a moderate alleviation between the 24 and 96 h observation time points indicating the worsening epidermal status of the animals. This phenomenon can be explained by the unbalanced function of two different components of the skin barrier: the physical barrier (stratum corneum thickening) and the immunological barrier (inflammatory cascade on IL23/Th17 axis). The reduced permeability at the later time point can be a consequence of keratinocyte proliferation, plaque formation and the consequently reduced transappendageal absorption of the test compound. These results indicate that the topical drug therapy has a better chance to effectively improve the symptoms and pathology of psoriasis at the early phase. Moreover, it is important to gain insights into the primary drug penetration pathways (transcellular, paracellular, transappendagaeal) in normal tissues before drug administration on the psoriatic skin to assist the right selection of medicinal products in an appropriate formulation.

## 4. Materials and Methods

### 4.1. Animals

The in vivo studies were conducted in female C57BL/6J, TRPV1 receptor gene knockout (KO, -/-) and TRPA1 KO mice (8–10 weeks, 20–25 g). The mice were bred and kept under standard pathogen-free (SPF) conditions in the Laboratory Animal House of the Department of Pharmacology and Pharmacotherapy of the University of Pécs, at 24–25 °C, provided with food and water ad libitum. All procedures were carried out according to the 1998/XXVIII Act of the Hungarian Parliament on Animal Protection and Consideration Decree of Scientific Procedures of Animal Experiments (243/1988). The animal study protocol was approved by the Ethics Committee on Animal Research of the University of Pécs, in full accordance with the Ethical Codex of Animal Experiments (license number: BA 02/2000-36/2017). After termination of in vivo experiments, the dorsal skins were excised and frozen at −20 °C until the ex vivo permeability studies.

### 4.2. Chemicals and Drug Formulations

Aldara cream was used for induction of psoriasiform dermatitis on shaved back skin of mice. It was purchased from Meda Pharma (Meda Pharma, Budapest, Hungary). The active ingredient of the Aldara cream is IMQ (5%). IMQ (imidazole-quinoline-amine) is a nucleotide-like small molecule and acts as an immune response modifier, it can activate certain cells of the immune system (mainly plasmacytoid dendritic cells and Langerhans cells in the skin) via toll-like receptor 7 and 8 (TLR7, 8) receptors located in the membrane of endosomes.

2% caffeine cream was used as a model formulation. Caffeine is a popular hydrophilic model drug; it is widely studied, easily available, and measurable with UV-VIS spectroscopy. It was purchased from Sigma (Sigma-Hungary Kft, Budapest, Hungary) and suspended in the following cream: 46% Vaseline ointment, 10% propylenglycol, 4% paraffin oil, 22% purified water. The components of the vaseline ointment were: 26% vaselinum album, 12% alcohol cetylstearylicum, 10% propylenglycol, 8% paraffin oil, 4% polysorbate, and 40% purified water.

In the microfluidic chip studies, an extracellular fluid-like perfusion solution, the artificial peripheral perfusion fluid (PPF), was used, which was composed of the following components: 147 mM NaCl, 4 mM KCl and 2.3 mM CaCl_2_·2 H_2_O. All ingredients were purchased from Sigma-Hungary Kft, Budapest, Hungary.

### 4.3. Induction of Psoriasiform Dermatitis in Mice

The hair of the animals was removed from the dorsal skin by shaving using an electric shaver, and with depilatory cream one day prior to the first Aldara or Vaseline treatment. The shaved back skin of C57BL/6J, TRPV1 KO and TRPA1 KO mice were treated with 62.5 mg Aldara cream daily for 4 days for induction of psoriasiform skin inflammation. In the control group, Vaseline was used in the same amount. All examinations were carried out under ketamine (100 mg/kg i.p., Richter, Hungary) and xylazine (5 mg/kg i.p., Lavet, Hungary) anesthesia. 24 and 96 h after the first Aldara or Vaseline treatment, the mice were sacrificed using cervical dislocation. Dorsal skin samples were then collected for drug diffusion study and microscopic analysis. Five animals were used in each group.

### 4.4. Measurement of Functional Parameters of Psoriasiform Skin Inflammation

Double-fold dorsal skin thickness was measured and averaged at two distinct sites using an engineer’s micrometer (Moore and Wright, Sheffield, England) with 0.1 mm accuracy, before starting the treatment (0 h, control measurement) and then prior to treatment with Aldara or control cream on each day of the experiments.

Blood perfusion was detected with the LASCA (LAser Speckle Contrast Analysis—PeriCam PSI System; Perimed, Sweden) method. Regions of interest (ROI) were selected according to the treated area on the dorsal skin. Mean perfusion of the treated area was generated by PimSoft software (Perimed, Sweden).

Transepidermal water loss (TEWL) was measured with a Tewameter TM Hex (Courage + Khazaka electronic GmbH, Cologne, Germany). TEWL value was expressed in units: g/m^2^/h. Three different skin sites were measured on the back skin of each mouse prior to the application of Aldara and control cream on each day of the experiment.

### 4.5. Ex Vivo Drug Diffusion Study on “Skin-On-A-Chip”

A vertical, flow-through diffusion cell has been developed by our research group, which is described in detail in previous papers [29,45]. It is a polydimethylsiloxane-based microfluidic chip, composed of three functional elements: on the top there is a donor chamber designed for holding the examined formulation, and on the bottom there is a receptor chamber. The integrated psoriatic or intact skin sample (originated from the in vivo experiments described at 2.3.) was placed between these compartments. The diffusion surface of the skin was 0.5 cm^2^, which was treated with 1000 µL of 2% caffeine cream using a Microman E piston gel pipette (Gilson, Middleton, WI, USA).

The PPF solution was loaded into a 5 mL syringe, the tube was connected to the microfluidic chip, and air bubbles were removed from the syringe, from the Teflon tubing and from the microchannel of the chip. Since this diffusion cell has a flow-through design, the fluid flow is continuous below the treated skin surface in the receptor compartment. The PPF solution was running through the chip, filling the microfluidic channel, the acceptor chambers and leaving the chip at the outlet into collection vials. Flow rate was controlled by a programmable syringe pump (NE-1000, New Era, Farmingdale, NY, USA) and kept at 4 µL/min during the experiments.

The samples were collected every 30 min. They were immediately placed onto dry ice and stored at −80 °C until spectrophotometric bioanalysis. The analysis was performed with a NanoDrop™ 2000 Spectrophotometer (ThermoFisher Scientific, Budapest, Hungary) at 273 nm.

### 4.6. Microscopic Evaluation of Ex Vivo Skin Samples

#### 4.6.1. Scanning Electron Microscopy (SEM)

Skin samples were fixed and dehydrated for analysis through scanning electron microscopy. Fresh skin samples were immersed in 10% *v*/*v* formaldehyde overnight and subsequently gradually dehydrated using a series of ethanol solutions as follows: 30% ethanol 2 h, 50% ethanol 2 h, 70% ethanol 2 h, 100% ethanol overnight (all % *v*/*v*). Before imaging, samples were removed from the solution, air dried, fixed on an aluminum sample holder stub using double sided adhesive carbon tape and finally the remaining solvent was removed in vacuum for 10 min. Images were acquired using a Hitachi TM4000Plus II scanning electron microscope (Fukuoka, Japan) using a 15 keV acceleration voltage in secondary electron mode (SE) at a 6 mm working distance.

#### 4.6.2. Classical Image Processing for SEM Image Analysis

During the study, the obtained images were also examined and analyzed using image processing methods in order to find the characteristics that help to distinguish between psoriatic and vaseline images recorded at different times. The initial assumption was that due to cell detachments, the size and the structure of the deeper intercellular regions will be different in several types of images, therefore, these differences were highlighted and quantified.

As a first step, based on the intensity values, a threshold (40 percent of the maximum intensity) was applied on the images to get binary images instead of grayscale images. After that, object search and counting were performed, where the objects were the gaps between the cells. The findContours method of the Open Source Computer Vision Library (OpenCV) was used to count the objects. This function uses Suzuki’s Contour tracing algorithm [46] to find the contours. By determining the gaps, the cells were also obtained and their total size in pixels was calculated. The following numerical values were assigned to each sample:

(1) $$N_{G}=\left|G\right|$$

where *G* is the set of gaps and the absolute value means the number of elements in the set.

(2) $$N_{P_{C}}=\left|P_{C}\right|$$

where *P_C_* is the set of pixels that do not belong to a gap but cells.

Two parameters were used, *N_G_* and *N_PC_*, to separate the different types of images. The achieved separation is described in the Results section.

For the different mouse strains tested, the ratio of cells to gaps was examined separately using N_G_ and N_PC_ values. For this purpuse, the images were divided into 250 × 250 images with a resolution of 1750 × 2560 pixels, so that a total of 60 images were obtained in each case. The results (*N_G_* and *N_PC_* values) are shown in Table 1 and Figure 6 and Figure 7.

#### 4.6.3. Two-Photon Microscopy

Two-photon images (see in the results section) were done using a laser scanning two-photon microscope (Femto2D-galvo, Femtonics, Budapest, Hungary). The microscope contained a 20× water immersion objective lens with 1.00 NA and 2 mm WD (XLUMPlanFL N, Olympus, Evident, Tokyo, Japan). The samples were excited at wavelenths between 830 and 920 nm with a femtosecond-pulsed two-photon laser (Chameleon, Coherent, Santa Clara, CA, USA) and the fluorescence was collected using two GaAsP photomultipliers (PMTs) for green and blue detection (H11706P-40, Hamamatsu, Herrsching am Ammersee, Germany). The figures were made with a MATLAB-based program (MES, Femtonics, Budapest, Hungary).

### 4.7. Statistical Evaluation of the Data

Statistical analysis was performed using Microsoft Excel 2010 (Microsoft Corporation) and GraphPad Prism 3.00 (GraphPad Software). All statistical comparisons were performed in Aldara-treated groups vs. vaseline-treated groups, using repeated measures or 2-way ANOVA followed by Bonferroni’s post hoc test or using Student’s *t* test. A *p* value of <0.05 was considered to indicate statistical significance. For image analysis, the Mann–Whitney U test was used for evaluation of the pixel differences between the healthy and psoriatic skins in SEM images.

## Figures and Tables

**Table 1:** Statistical evaluation of the scanning electronmicroscopic pictures after computerized image analysis using the Mann–Whitney test for comparison of control (VAS) and psoriatic (IMQ) skins in regard to the number of gaps (N_G_) and number of pixels in cells (N_PC_). The *p* values < 0.05 are considered as statistically significant differences.

Ples (VAS vs. IMQ)	N_G_ *p*-Value	N_PC_ *p*-Value
24 h WT	6.14 × 10^−7^	0.0299
96 h WT	0.037	0.0001
24 h TRPA1	0.486	0.48
96 h TRPA1	0.00167	8.58 × 10^−7^
24 h TRPV1	0.28	0.129
96 h TRPV1	0.00047	0.0022

**Table 2:** The main endogenous fluorophore component of excised skins.

Fluorophore	Emission Wavelength [nm]	Blue/Green
FADs	490–650	Blue and Green
Keratin	450–550	Blue and Green
Flavoprotein	460–480	Blue
Lipoamide dehydrogenase	510–550	Green

## Data Availability

The experimental data are available in the computer archives of the research laboratories.
